# Author Correction: Interstitial-fluid shear stresses induced by vertically oscillating head motion lower blood pressure in hypertensive rats and humans

**DOI:** 10.1038/s41551-023-01152-9

**Published:** 2023-11-07

**Authors:** Shuhei Murase, Naoyoshi Sakitani, Takahiro Maekawa, Daisuke Yoshino, Kouji Takano, Ayumu Konno, Hirokazu Hirai, Taku Saito, Sakae Tanaka, Keisuke Shinohara, Takuya Kishi, Yuki Yoshikawa, Takamasa Sakai, Makoto Ayaori, Hirohiko Inanami, Koji Tomiyasu, Atsushi Takashima, Toru Ogata, Hirotsugu Tsuchimochi, Shinya Sato, Shigeyoshi Saito, Kohzoh Yoshino, Yuiko Matsuura, Kenichi Funamoto, Hiroki Ochi, Masahiro Shinohara, Motoshi Nagao, Yasuhiro Sawada

**Affiliations:** 1 Department of Rehabilitation for Motor Functions, National Rehabilitation Center for Persons with Disabilities, Tokorozawa, Japan; 2 Department of Orthopaedic Surgery, Graduate School of Medicine, The University of Tokyo, Tokyo, Japan; 3 Department of Cell Biology, National Cerebral and Cardiovascular Center, Suita, Japan; 4 Division of Advanced Applied Physics, Institute of Engineering, Tokyo University of Agriculture and Technology, Koganei, Japan; 5 Department of Rehabilitation for Brain Functions, National Rehabilitation Center for Persons with Disabilities, Tokorozawa, Japan; 6 Department of Neurophysiology & Neural Repair, Gunma University Graduate School of Medicine, Maebashi, Japan; 7 Department of Cardiovascular Medicine, Faculty of Medical Sciences, Kyushu University, Fukuoka, Japan; 8 Department of Cardiology, Graduate School of Medicine, International University of Health and Welfare, Okawa, Japan; 9 Department of Chemistry and Biotechnology, Graduate School of Engineering, The University of Tokyo, Tokyo, Japan; 10 Tokorozawa Heart Center, Tokorozawa, Japan; 11 Inanami Spine & Joint Hospital/Iwai Orthopaedic Medical Hospital, Iwai Medical Foundation, Tokyo, Japan; 12 Center of Sports Science and Health Promotion, National Rehabilitation Center for Persons with Disabilities, Tokorozawa, Japan; 13 Department of Assistive Technology, National Rehabilitation Center for Persons with Disabilities, Tokorozawa, Japan; 14 Department of Rehabilitation Medicine, Gngraduate School of Medicine, The University of Tokyo, Tokyo, Japan; 15 Department of Cardiac Physiology, National Cerebral and Cardiovascular Center, Suita, Japan; 16 Department of Advanced Medical Technologies, National Cerebral and Cardiovascular Center, Suita, Japan; 17 Department of Medical Physics and Engineering, Division of Health Sciences, Osaka University Graduate School of Medicine, Suita, Japan; 18 School of Biological and Environmental Sciences, Kwansei Gakuin University, Sanda, Japan; 19 Department of Health and Sports, Niigata University of Health and Welfare, Niigata, Japan; 20 Institute of Fluid Science, Tohoku University, Sendai, Japan; 21 Department of Clinical Research, National Rehabilitation Center for Persons with Disabilities, Tokorozawa, Japan

**Keywords:** Biomedical engineering, Cardiovascular biology

Correction to: *Nature Biomedical Engineering* 10.1038/s41551-023-01061-x, published online 6 July 2023.

This article was originally published under a standard Springer Nature license (© The Author(s), under exclusive license to Springer Nature Limited 2023). It is now available as an open-access paper under a Creative Commons Attribution 4.0 International license, © The Author(s). The error has been corrected in the HTML and PDF versions of the article.

