# Supplementary material for: Interstitial-fluid shear stresses induced by vertically oscillating head motion lower blood pressure in hypertensive rats and humans
Source: Nat Biomed Eng. 2023 Jul 6;7(11):1350–73. doi: 10.1038/s41551-023-01061-x (PMC10651490; doi:10.1038/s41551-023-01061-x)
Supplement: Supplementary file 1 — Supplementary figures, tables, details on statistics, video captions and references. [file 41551_2023_1061_MOESM1_ESM.pdf]

nature biomedical engineering

Article https://doi.org/10.1038/s41551-023-01061-x

Interstitial-fluid shear stresses induced by vertically oscillating head motion lower blood pressure in hypertensive rats and humans

In the format provided by the authors and unedited

Contents

1. Supplementary Figure 1 | Hydrogel introduction in the RVLM does not affect the pressor and depressor responses to AngII and valsartan, respectively, in SHRSPs.
2. Supplementary Figure 2 | Hydrogel introduction to the RVLM eliminates the antihypertensive effect of treadmill running in SHRSPs.
3. Supplementary Figure 3 | Hydrogel introduction does not affect the cell number/apoptosis, the expression of pro-inflammatory cytokines, and the pressure in the RVLM of SHRSPs.
4. Supplementary Figure 4 | BP, HR, and aortic depressor nerve activity (ADNA) remain unchanged during the transition from before to after the initiation of PHM in SHRSPs.
5. Supplementary Figure 5 | Vertically oscillating chair that reproduces the mechanical accelerations in the head during light jogging, and the design and results of protocol 1 as a pilot study to examine antihypertensive effect of VOCR in hypertensive adult humans.
6. Supplementary Figure 6 | Design and blood test results of the human study of protocol 2.
7. Supplementary Figure 7 | Design of the human study of protocol 3, and sex-segregated analysis of BP and HR in protocols 2 and 3.
8. Supplementary Figure 8 | Schematic representation of the antihypertensive effects observed in PHM of hypertensive rats and VOCR of hypertensive humans.
9. Supplementary Table 1 | Calculation of the magnitude of PHM-generated FSS on rat RVLM cells.

10. Supplementary Table 2 | Information of participants in the human studies of protocol 1 and 2.
11. Supplementary Table 3 | Information of participants in the study of protocol 3.
12. Description of Supplementary Videos
13. References
14. Supplementary Data 1 | Uncropped blots for Supplementary Figure 3h.
15. Information of Statistical Analyses

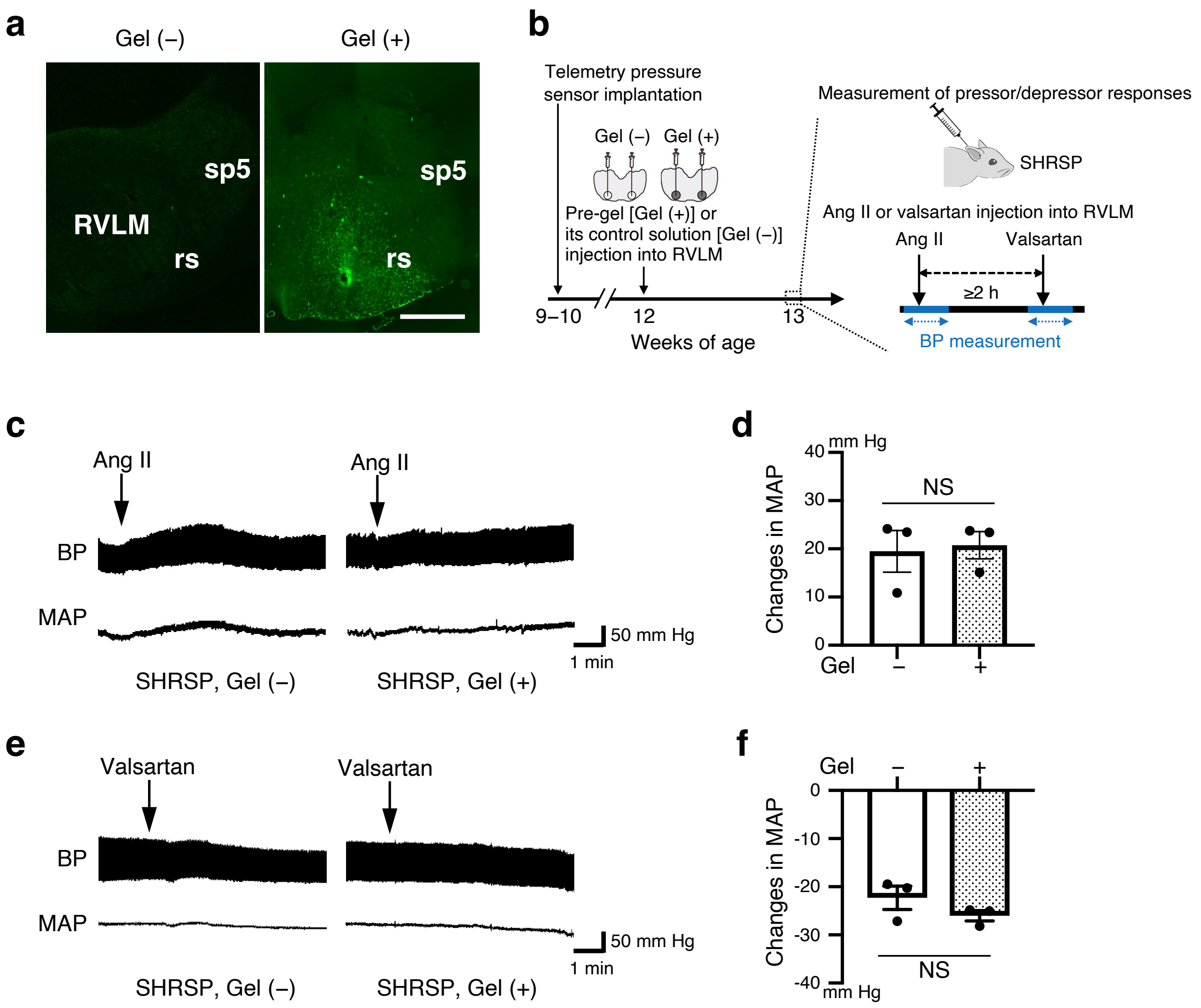

Supplementary Fig. 1 | Hydrogel introduction in the RVLM does not affect the pressor and depressor responses to AngII and valsartan, respectively, in SHRSPs. a, Introduction of the PEG hydrogel in the rat RVLM. Twenty-four hours after the injection of control ungelatable fluorescent PEG solution (left) or one week after the injection of pre-gel fluorescent PEG solution (right), brainstem samples were prepared. Coronal-section images representative of three rats with similar results are shown. Scale bar, 1 mm. **b–f,** Pressor and depressor responses analyzed one week after the injection of pre-gel PEG solution or its ungelatable control. **(b)** Schematic representation of the experimental protocol. Pressor and depressor responses were

analyzed as in Fig. 2. **(c–f)** Representative trajectories **(c,e)** and quantification **(d,f)** of the BP ascent upon AngII injection **(c,d)** and descent upon valsartan injection **(e,f)** to the RVLM of SHRSPs with or without the hydrogel introduction (**d**: $P = 0.8226$. **f**: $P = 0.2342$. $n = 3$ rats for each group). Right-angled scale bars, 1 min / 50 mm Hg. Data are presented as mean ± s.e.m. NS, not significant; unpaired two-tailed Student's *t*-test.

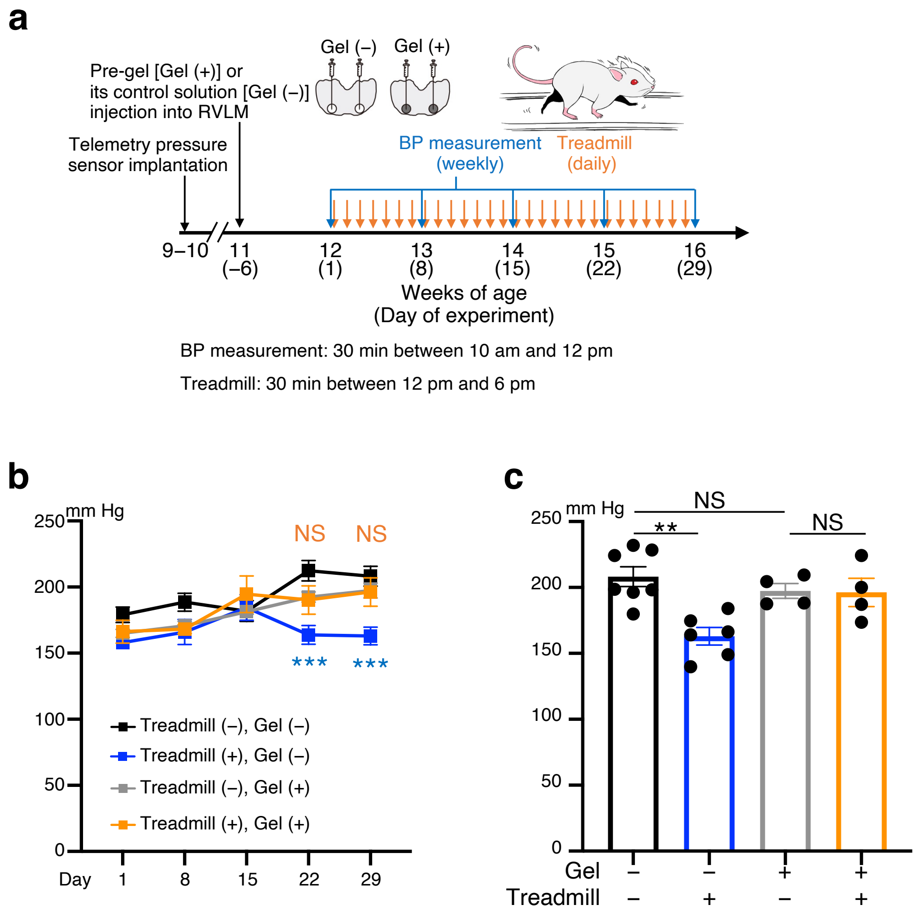

Supplementary Fig. 2 | Hydrogel introduction to the RVLM eliminates the antihypertensive effect of treadmill running in SHRSPs. a, Schematic representation of the experimental protocol to analyze the effects of treadmill running on the BP in SHRSPs. **b,c,** Time courses **(b)** and values on Day 29 **(c)** of MAP in SHRSPs with (**b**: gray and orange lines, **c**: columns 3 and 4) and without (**b**: black and blue lines, **c**: columns 1 and 2) the hydrogel introduction, subjected to either daily treadmill running (30 min/day, 28 days; **b**: blue and orange lines, **c**: columns 2 and 4) or its control (placing on the belt without turning on the treadmilling; **b**: black and gray lines, **c**: columns 1 and 3) [**b**, black vs. blue: $P > 0.9999$ for Day 15, $P = 0.0001$ for Day 22, $P = 0.0004$ for Day 29; gray vs. orange: $P > 0.9999$ for Day 15, Day

22, and Day 29. **c**: $P = 0.0015$ for column 1 vs. 2, $P = 0.9997$ for column 3 vs. 4, $P = 0.7751$ for column 1 vs. 3. $n = 7$ rats for treadmill (–)/Gel (–); $n = 6$ rats for treadmill (+)/Gel (–); $n = 4$ rats for each group of Gel (+)]. Data are presented as mean ± s.e.m. $^{**}P < 0.01$, $^{***}P < 0.001$; NS, not significant; two-way repeated measures ANOVA with Bonferroni's post hoc comparisons test **(b)** or one-way ANOVA with Tukey's post hoc multiple comparisons test **(c)**.

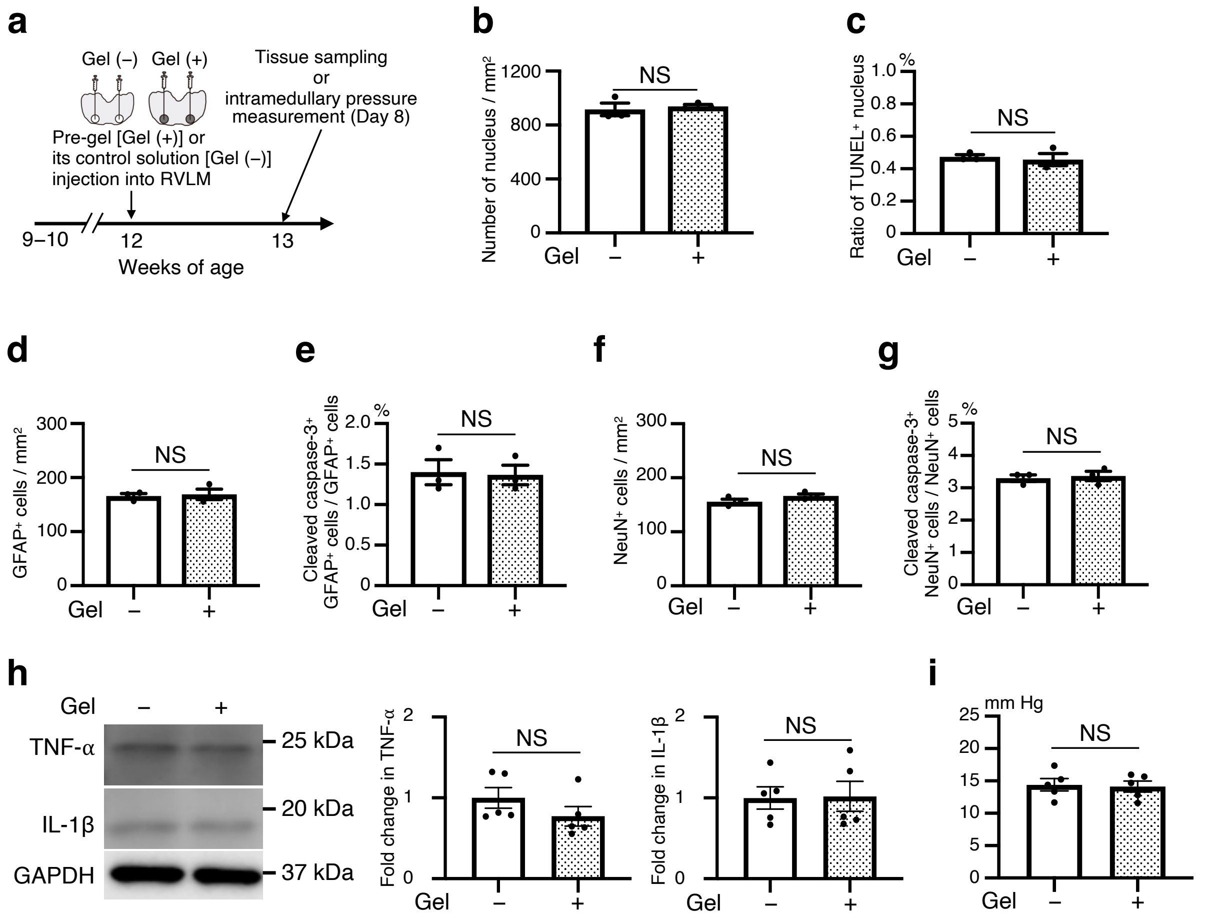

Supplementary Fig. 3 | Hydrogel introduction does not affect the cell number/apoptosis, the expression of pro-inflammatory cytokines, and the pressure in the RVLM of SHRSPs.

a, Schematic representation of the experimental protocol to analyze the hydrogel-introduced RVLM in SHRSPs. **b–g,** Effects of hydrogel introduction on the survival **(b,d,f)** and apoptosis **(c,e,g)** of total cells **(b,c)**, astrocytes **(d,e)**, and neurons **(f,g)** in the RVLM of SHRSPs. Fixed rat RVLM sections were subjected to TUNEL assay **(b,c)**, or combinations of anti-GFAP for glial fibrillar acidic protein-positive astrocytes, anti-NeuN for mature neurons, and anti-cleaved caspase-3 for apoptotic cells immunostaining **(e–g)**. DAPI-positive nuclei **(b)**, GFAP- **(d)** or NeuN- **(f)** positive cells were counted, and the relative populations of cells doubly positive for

indicated combinations of TUNEL **(c)** and cleaved caspase-3 **(e,g)** were quantified. Each value in **(b−g)** represents an average from five images of 1 x 1-mm area analyzed for each rat. **h,** Expression of TNF-α and IL-1β in SHRSPs' RVLM with and without the hydrogel introduction. Anti-TNF-α, anti-IL-1β, and anti-GAPDH immunoblots of samples prepared from two individual rats with (lane 2) and without (lane 1) hydrogel introduction (left). Expressions of TNF-α (center) and IL-1β (right) were normalized against GAPDH expression, and scaled with the mean values of the control samples [Gel (−)] set as 1. **i,** Intramedullary pressure of SHRSPs with and without the hydrogel introduction in their RVLM. The intramedullary pressure was measured as in Fig. 4a−d, and the mean value was obtained from 30-s steady-state continuous measurement for each rat (**b**: $P = 0.6518$. **c**: $P = 0.6943$. **d**: $P = 0.7938$. **e**: $P = 0.8722$. **f**: $P = 0.1679$. **g**: $P = 0.7247$. **h**: $P = 0.2312$ for TNF-α, $P = 0.9342$ for IL-1β. **i**: $P = 0.8433$. **b-g**: $n = 3$ rats for each group. **h,i**: $n = 5$ rats for each group). Data are presented as mean ± s.e.m. NS, not significant; unpaired two-tailed Student's *t*-test **(b-i)**.

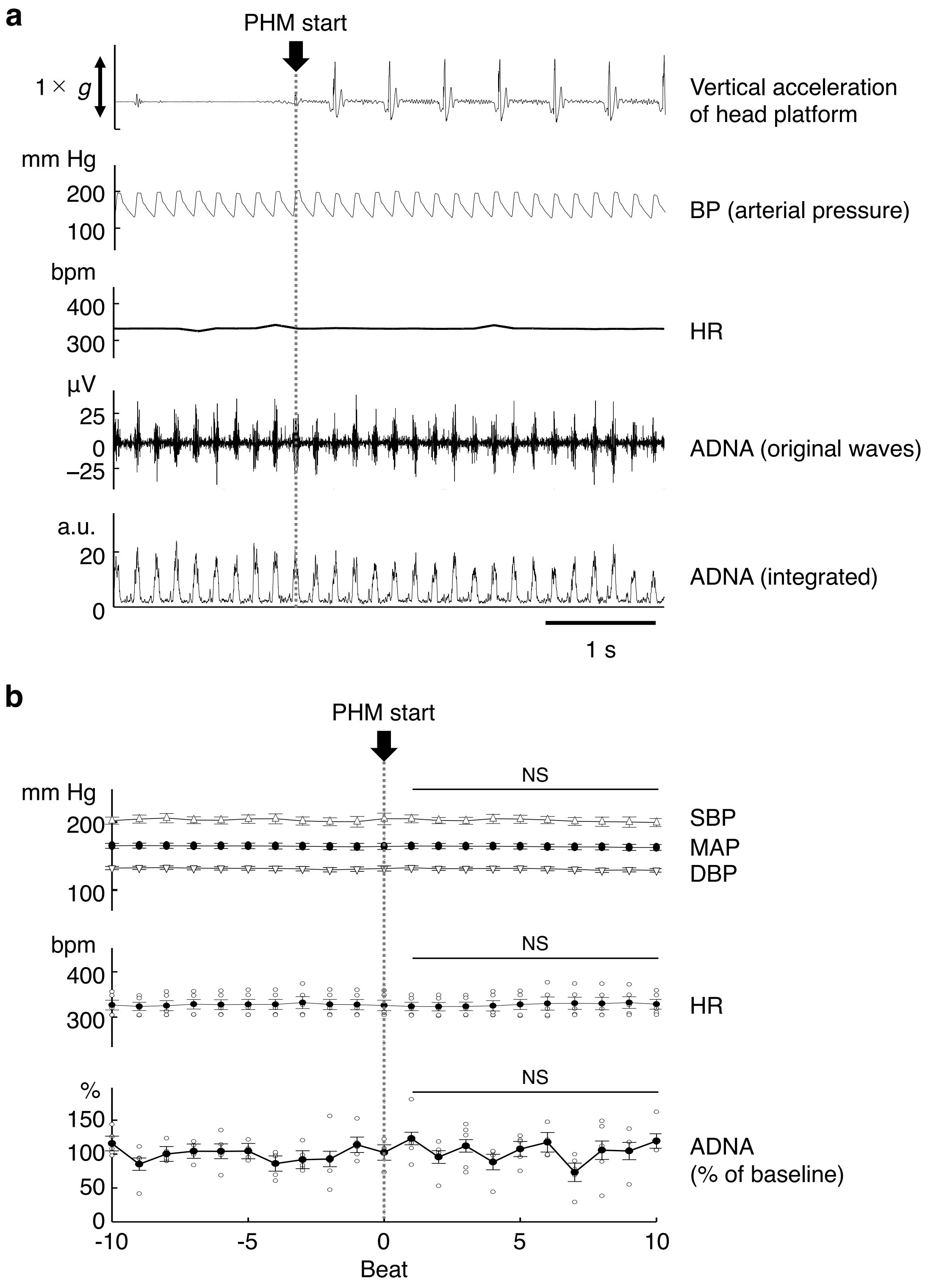

Supplementary Fig. 4 | BP, HR, and aortic depressor nerve activity (ADNA) remain unchanged during the transition from before to after the initiation of PHM in SHRSPs. a, BP, HR, and ADNA in 12−16-week-old male SHRSPs monitored and recorded simultaneously

with vertical accelerations of the oscillating PHM platform. Integrated ADNA was normalized in each rat and is presented in arbitrary units (a.u.) (see Methods). Data shown represent five biologically independent experiments using five different SHRSPs with similar results. **b,** Beat-by-beat BP (SBP, MAP, and DBP), HR, and ADNA. The beat that elicited SBP immediately before the PHM initiation was defined as "beat 0". Statistical analysis was conducted against the mean values of beat –9 to beat 0 (n = 5 rats). Data are presented as mean ± s.e.m. NS, not significant; one-way repeated measures ANOVA with Dunnett's multiple comparisons test. Arrows and dotted lines in (**a,b**) indicate the time point of PHM initiation. Details of statistical analyses are provided in the Information of Statistical Analyses section.

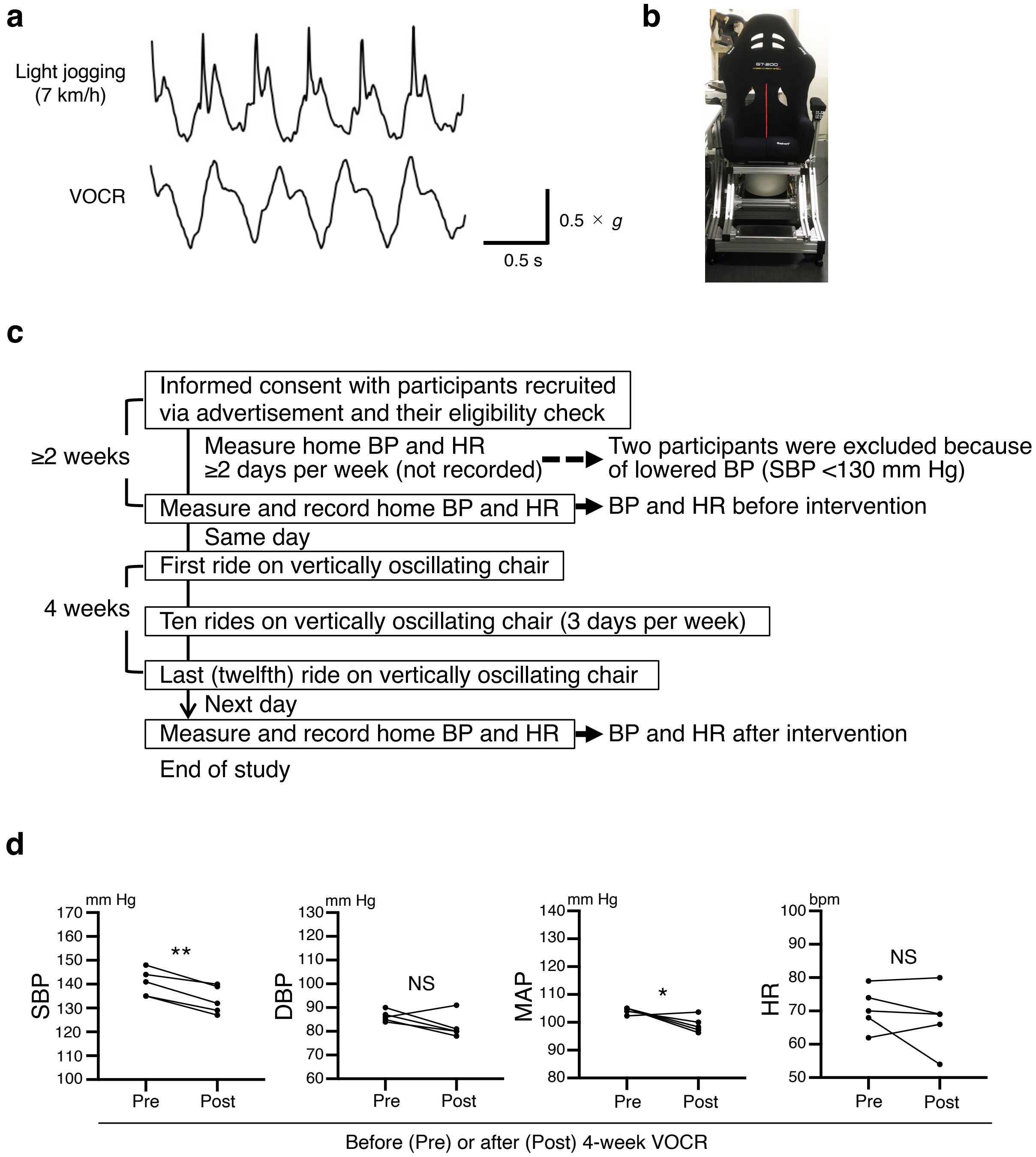

Supplementary Fig. 5 | Vertically oscillating chair that reproduces the mechanical accelerations in the head during light jogging, and the design and results of protocol 1 as a pilot study to examine antihypertensive effect of VOCR in hypertensive adult humans. a, Vertical accelerations generated at adult human head during light jogging on a treadmill machine (velocity: 7 km/h) and VOCR (frequency: 2 Hz). The VOCR system was adjusted to

produce ~1.0 × *g* vertical acceleration peaks. Right-angled scale bar, 0.5 s / 0.5 × *g*. Images are representative of three biologically independent experiments with similar results. **b**, Photograph of the chair. **c,** Schematic representation of protocol 1. **d,** From left to right, SBP, DBP, MAP, and HR "value of the day"s immediately before and after 4-week VOCR in the study of protocol 1 (SBP: $P = 0.0018$. DBP: $P = 0.1509$. MAP: $P = 0.0459$. HR: $P = 0.3900$. $n = 5$). *$P < 0.05$; **$P < 0.01$; NS, not significant; paired two-tailed Student's *t*-test.

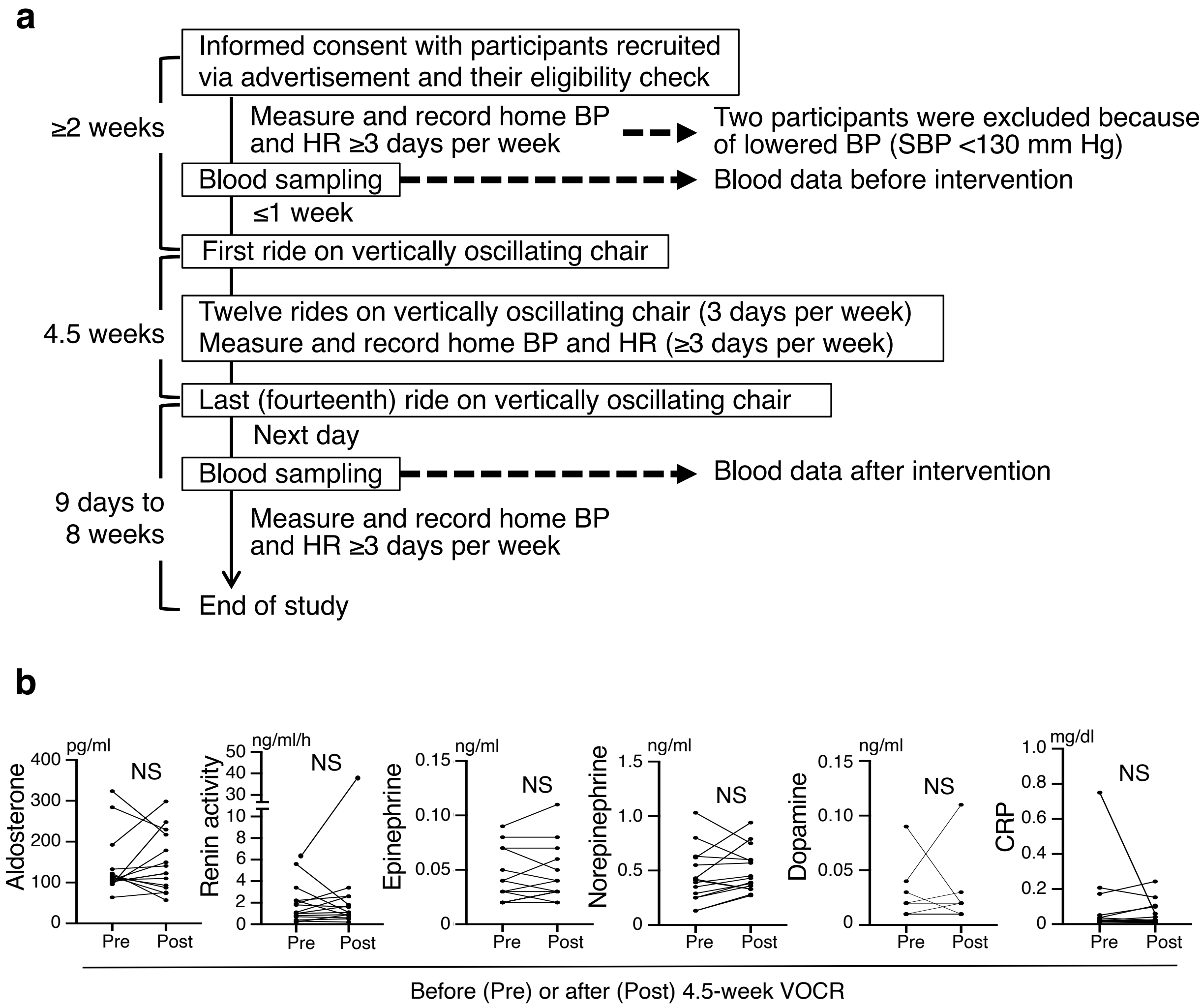

Supplementary Fig. 6 | Design and blood test results of the human study of protocol 2. a, Schematic representation of protocol 2. **b,** Blood test values before and after the intervention period (from left to right, aldosterone, renin activity, epinephrine, norepinephrine, dopamine, and CRP). Significant change was not observed in any of tested parameters. NS, not significant; paired two-tailed Student's *t*-test (Aldosterone: $P = 0.6265$. Renin activity: $P = 0.3794$. Epinephrine: $P = 0.5103$. Norepinephrine: $P = 0.2653$. Dopamine: $P > 0.9999$. CRP: $P = 0.4412$. $n = 15$). A participant (participant #18) showed a large increase in plasma renin activity after VOCR. We advised him to consult his primary care physician, who ruled out the

disqualifying conditions for this study (e.g., severe renal disease; see Methods) based on comprehensive evaluation. Therefore, we did not exclude participant #18 from our statistical analysis of BP and HR.

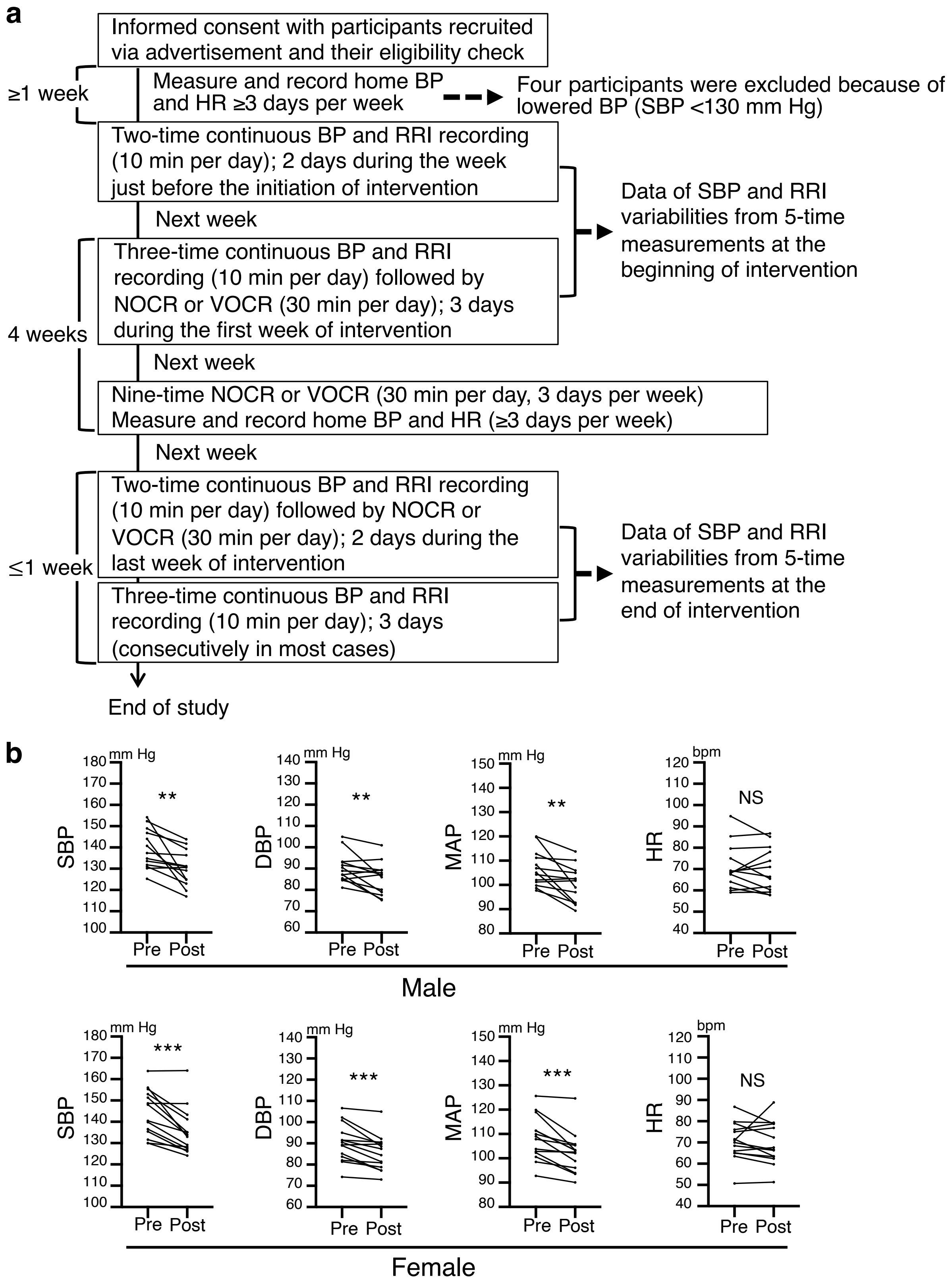

Supplementary Fig. 7 | Design of the human study of protocol 3, and sex-segregated analysis of BP and HR in protocols 2 and 3. **a,** Schematic representation of protocol 3. **b,**

From left to right, SBP, DBP, MAP, and HR "value of the week"s immediately before and after 4.5-week VOCR in the study of protocols 2 and 3. To avoid duplicate inputs, the VOCR data from participants #22 and #31 in protocol 3, who were participants #15 and #7 in protocol 2 (see Supplementary Table 3), were excluded from this analysis (male, SBP: $P = 0.0033$. DBP: $P = 0.0094$. MAP: $P = 0.0046$. HR: $P = 0.4373$. $n = 13$; female, SBP: $P = 0.0002$. DBP: $P = 0.0005$. MAP: $P = 0.0003$. HR: $P = 0.6257$. $n = 14$). **$P < 0.01$; ***$P < 0.001$; NS, not significant; paired two-tailed Student's *t*-test.

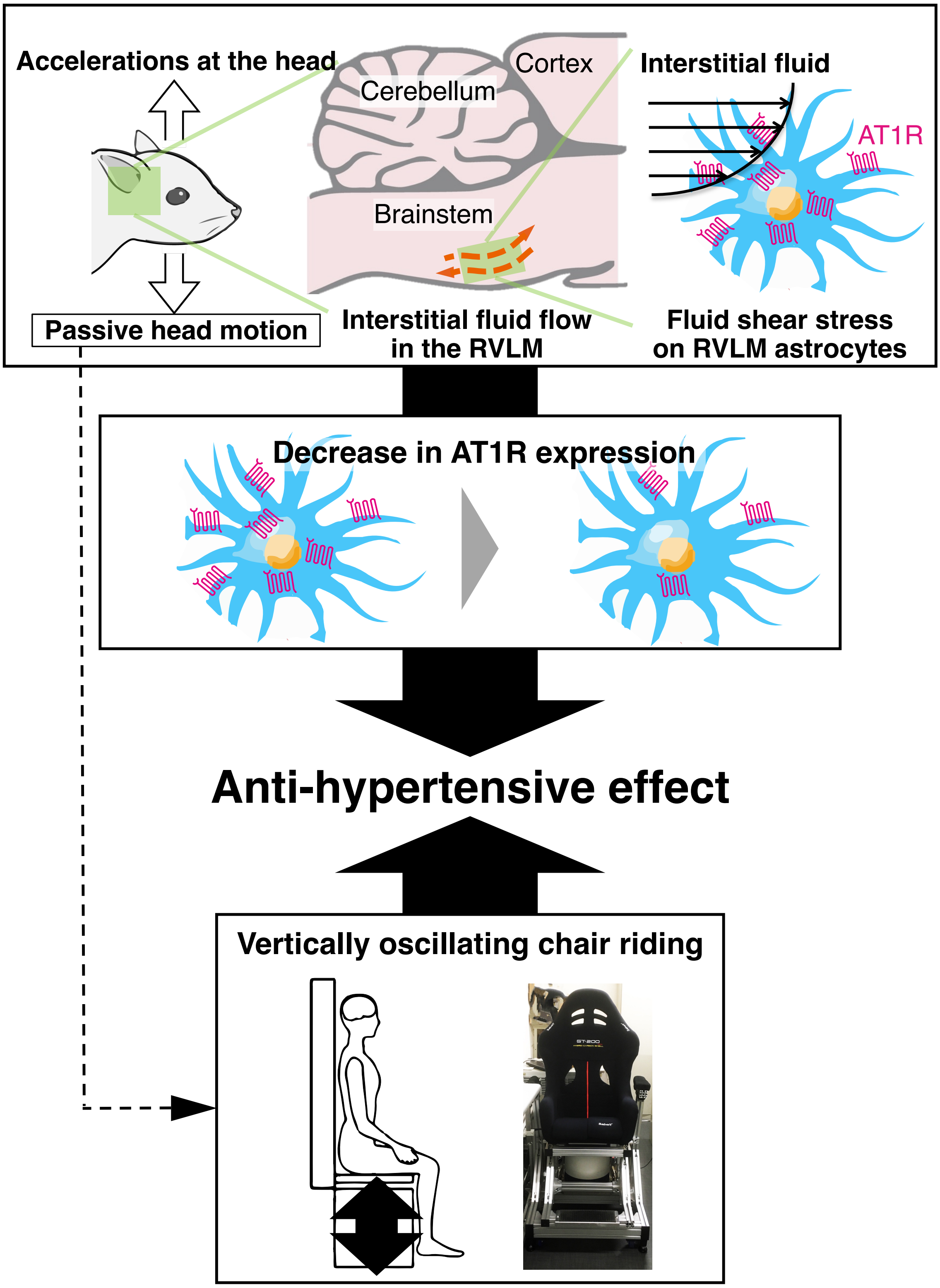

Supplementary Fig. 8 | Schematic representation of the antihypertensive effects observed in PHM of hypertensive rats and VOCR of hypertensive humans. The results from our

animal experiments indicate that cyclical application of mechanical intervention to the head generates interstitial fluid movement in the RVLM, leading to FSS-induced decrease in the AT1R expression in astrocytes in situ, and thereby ameliorates hypertension. Our studies also show that the VOCR of hypertensive adult humans, which produces vertical accelerations at their heads, lowers their BP.

a

Property	Value
Porosity (ε)	0.2 (20%)
Diameter of interstitial space (D_p; µm)	0.10–0.48
Kozeny constant (k)	4.5–5.5
Viscosity of interstitial fluid (μ; mPa•s)	0.72
Velocity of interstitial fluid movement (flow) (µm/s)	0.4–0.6

b

FSS (τ_x) at the cell surface:

$$\tau = \frac{\mu u_\infty}{\sqrt{K_p}}$$

$$K_p = \frac{D_p^2 \varepsilon^3}{36k(1-\varepsilon)^2}$$

, where K_p is the Darcy permeability in the brain (RVLM).

When the values listed in **a** are introduced in these equations, the Darcy permeability is calculated as $\mathbf{6.63 \times 10^{-19} - 1.45 \times 10^{-17}\ m^2}$, and the magnitude of FSS is estimated as **0.076–0.53 Pa**.

Supplementary Table 1 | Calculation of the magnitude of PHM-generated FSS on rat RVLM cells. a, Values referenced for calculation of the magnitude of FSS that PHM generated in the rat RVLM. Based on the estimated structure of the interstitial space in the rat RVLM (Extended Data Fig. 6e and Extended Data Fig. 7d), we referenced 4.5–5.5 as the Kozeny constant[1-3] to calculate the Darcy permeability (see Methods). Because the velocity of interstitial fluid movement has been reported to be ~0.2 µm/s in sedentary rats[4] and mice[5], we entered 0.4–

0.6 μm/s as its value during PHM, based on our interpretation of the μCT study (i.e., two- to three-fold increase in velocity; Fig. 4g,h). The property of the interstitial fluid in the brain, whose composition is similar to that of the cerebrospinal fluid (CSF)[6], was referenced from previous studies describing the viscosity of human CSF[7,8]. The diameter of the individual interstitial space was referenced from the values of the cross-sectional area of the interstitial space as shown in Extended Data Fig. 7c. **b,** Calculation of the magnitude of FSS generated by PHM. The Darcy permeability (K_p) is calculated as $6.63 \times 10^{-19} – 1.45 \times 10^{-17}$ m^2. Following the calculation reported previously[9], the magnitude of FSS (τ) at the cell surface is estimated as 0.076–0.53 Pa.

Participant #	Sex	Age (years)	Body weight (kg)	Height (cm)	BMI	Period since diagnosis or self-recognition as hypertension	Declared health problems and diseases other than hypertension	Smoking	Alcohol (if yes, how often)	Current medication (dose per day)	Habitual exercise (times per week)	SBP/DBP (mm Hg) and HR (bpm) just before (left)	and after (right)	VOCR period (month of first - last bout of VOCR)
1	Male	60	73	165.2	27.2	13 years	Hyperuricemia	No	Almost every day	Azilsartan ・Amlodin (20 mg • 5 mg)<br>Febuxostat (10 mg)<br>Bisoprolol fumarate (5 mg)	Walking<br>90 min<br>(3 or 4 times)	148/85<br>62	139/78<br>66	February - March
2	Female	53	50	159	19.8	14 years	None	No	Almost every day	None	Sit-ups 2 x 30 times<br>(every day)	135/90<br>70	127/81<br>69	March
3	Male	37	113	186	32.7	19 years	None	No	Occasionally	None	Judo<br>(once)	135/86<br>68	129/91<br>54	March
4	Female	60	73	156	30	6 years	None	No	Occasionally	None	None	141/87<br>79	132/80<br>80	April - May
5	Female	52	50	148	22.8	1 year	None	No	Occasionally	Azilsartan (20 mg)<br>Amlodin (2.5 mg)	None	144/84<br>74	140/80<br>69	April - May
6	Female	57	68	164	25.3	10 years	None	No	Occasionally	None	None	130/74<br>79	124/73<br>72	November - December
7	Male	61	67	168	23.7	Uncertain (<1 year)	Diabetes mellitus<br>Hyperlipidemia	No	No	Metformin hydrochloride (1000 mg)<br>Rosuvastatin calcium (5 mg)<br>Sitagliptin phosphate hydrate (50 mg)	Walking<br>120 min<br>(once)	137/92<br>85	136/89<br>87	November - December
8	Male	46	72	178	22.7	3 months	Allergic rhinitis	Yes	Occasionally	Montelukast sodium (10 mg)<br>Ebastine (10 mg)	Walking<br>60 min<br>(once)<br>Stretching<br>30 min<br>(once)	125/86<br>68	117/76<br>60	November - December
9	Male	55	87	173	29.1	7 months	Diabetes mellitus	No	No	None	Karate<br>(2 or 3 times)	150/85<br>72	146/87<br>71	November - December
10	Male	70	62	166	22.5	6 months	None	No	Occasionally	None	Walking<br>40 min<br>(4 times)	144/91<br>80	120/79<br>80	November - December
11	Female	60	45	157	18.3	Uncertain (>1year)	Breast cancer<br>(post-surgery)	No	Almost every day	Tamoxifen citrate (20 mg)	None	153/91<br>72	141/84<br>64	November - December
12	Male	68	72	171	24.6	Uncertain (<1 year)	None	No	No	None	None	134/85<br>69	131/87<br>71	January - February
13	Female	56	58	160	22.7	7 years	None	No	No	Telmisartan • Amlodipine<br>(40 mg • 5 mg)	None	140/95<br>66	129/90<br>68	January - February
14	Male	49	69	168	24.5	Uncertain (<1 year)	Diabetes mellitus	No	No	Sitagliptin phosphate hydrate (50 mg)<br>Acetazolamide (250 mg)	None	130/85<br>95	131/80<br>85	January - February

Participant #	Sex<br>Age (years)<br>Body weight (kg)<br>Height (cm)<br>BMI	Period since diagnosis or self-recognition as hypertension<br>Declared health problems and diseases other than hypertension	Smoking<br>Alcohol (if yes, how often)	Current medication (dose per day)	Habitual exercise (times per week)	SBP/DBP (mm Hg) and HR (bpm) just before (left) and after (right) VOCR period (month of first - last bout of VOCR): Before (left)	After (right)	Month of first - last bout of VOCR
15	Male<br>43<br>69<br>165<br>25.3	Uncertain (<1 year)<br>None	No<br>Occasionally	None	None	141/87<br>75	125/75<br>64	August - September
16	Female<br>48<br>45.3<br>157.2<br>18.3	15 years<br>None	No<br>Occasionally	None	Walking<br>60 min<br>(6 times)	156/101<br>71	133/89<br>89	October - November
17	Male<br>65<br>60.1<br>167.1<br>21.5	8 years<br>None	Yes<br>Almost every day	None	None	135/89<br>59	131/88<br>59	October
18	Male<br>55<br>93.4<br>168.1<br>33.1	8 years<br>Reflex esophagitis<br>Hyperlipidemia	No<br>Almost every day	Azilsartan (20 mg)<br>Lansoprazole (15 mg)<br>Atorvastatin calcium hydrate (10 mg)<br>Benidipine hydrochloride (4 mg)	None	149/105<br>68	139/101<br>78	November - December
19	Male<br>41<br>92.3<br>183.2<br>27.5	2 years<br>None	No<br>Almost every day	None	Walking<br>60 min<br>(3 times)	132/87<br>61	129/89<br>58	January - February
20	Female<br>65<br>48.7<br>158.6<br>19.4	1 year<br>Hyperlipidemia<br>Lumbar spinal canal stenosis (post-surgery)	No<br>Almost every day	Amlodipine besilate (5 mg)<br>Atorvastatin calcium hydrate (10 mg)<br>Limaprost alfadex (15 μg)<br>Loxoprofen sodium hydrate (120 mg)<br>Rebamipide (200 mg)	Swimming<br>40 min<br>(5 times)	135/84<br>70	126/77<br>67	January - February
21	Male<br>32<br>65.1<br>168.9<br>22.8	2 years<br>None	No<br>Almost every day	None	None	154/102<br>68	125/86<br>74	January - February

Supplementary Table 2 | Information of participants in the human studies of protocol 1 and 2. SBP/DBP (mm Hg) and HR (bpm) just before and after VOCR are "value of the day"s in participants #1−#5 (protocol 1) and "value of the week"s in participants #6−#21 (protocol 2). Participant 9 was excluded from our statistical analysis because of the high serum CRP value before VOCR (2.85 mg/dL), which made it difficult to rule out acute infection, a possible disqualifier, at the time of the initiation of VOCR, albeit the lack of specific complaint or local symptom related to acute physical problem(s). His serum CRP after the VOCR period was within the normal range (0.12 mg/dL).

Participant #	Sex<br>Age (years)<br>Body weight (kg)<br>Height (cm)<br>BMI	Period since diagnosis or self-recognition as hypertension<br>Declared health problems and diseases other than hypertension	Smoking<br>Alcohol (if yes, how often)	Current medication (dose per day)	Habitual exercise (times per week)	SBP/DBP (mm Hg) and HR (bpm) just before (left) and after (right) NOCR period (month of first - last bout of NOCR)		SBP/DBP (mm Hg) and HR (bpm) just before (left) and after (right) VOCR period (month of first - last bout of VOCR)	
22<br>(15)	Male<br>44<br>69<br>165<br>25.3	Uncertain (>1 year)<br>None	No<br>Occasionally	None	None	132/84<br>69	136/85<br>70	134/84<br>70	124/73<br>69
						September - October		March - April	
23	Female<br>69<br>38<br>152<br>16.5	3 years<br>Lumbar spinal canal stenosis	No<br>No	Mecobalamin (1.5 mg)	Walking<br>30 min<br>(2 or 3 times)	141/82<br>62	148/89<br>65	148/89<br>65	134/81<br>63
						October - November		November - December	
24	Female<br>45<br>49<br>157<br>19.9	 None	No<br>Almost every day	None	Walking<br>30 min<br>(once or twice)	144/94<br>86	150/96<br>79	155/102<br>87	143/92<br>79
						October - November		March - April	
25<br>(11)	Female<br>61<br>45<br>157<br>18.3	Uncertain (>1 year)<br>Breast cancer (post-surgery)	No<br>Almost every day	Tamoxifen citrate (20 mg)	None	150/96<br>83	139/91<br>89	ND	ND
						October - November		ND	
26<br>(17)	Male<br>66<br>60.1<br>167.1<br>21.5	8 years<br>None	Yes<br>Almost every day	None	None	132/87<br>58	129/85<br>60	ND	ND
						October - December		ND	
27	Male<br>37<br>82<br>178<br>25.9	This study (<1 month)<br>None	No<br>Occasionally	None	None	142/92<br>63	142/94<br>65	ND	ND
						October - November		ND	
28	Male<br>23<br>125<br>178<br>39.5	This study (<1 month)<br>None	No<br>Occasionally	None	Resistance training<br>(3 times)	131/76<br>59	131/81<br>60	131/81<br>60	123/78<br>62
						November - December		December - January	
29	Female<br>59<br>77.5<br>160<br>30.3	14 years<br>Hyperlipidemia	No<br>No	Amlodin • Atorvastatin (5 mg • 5 mg)<br>Telmisartan (40 mg)<br>Vonoprazan (10 mg)	None	137/88<br>83	143/93<br>72	140/92<br>75	135/91<br>77
						November - December		January - February	
30<br>(20)	Female<br>66<br>48.7<br>158.6<br>19.4	2 years<br>Hyperlipidemia<br>Lumbar spinal canal stenosis (post-surgery)	No<br>Almost every day	Amlodipine besilate (5 mg)<br>Atorvastatin calcium hydrate (10 mg)<br>Limaprost alfadex (15 µg)<br>Loxoprofen sodium hydrate (120 mg)<br>Rebamipide (200 mg)	Swimming<br>40 min<br>(5 times)	129/80<br>67	125/77<br>70	ND	ND
						November - December		ND	
31<br>(7)	Male<br>62<br>67<br>168<br>23.7	Uncertain (>1 year)<br>Diabetes mellitus<br>Hyperlipidemia	No<br>No	Metformin hydrochloride (1000 mg)<br>Rosuvastatin calcium (5 mg)<br>Sitagliptin phosphate hydrate (50 mg)	Walking<br>120 min<br>(once)	141/86<br>87	148/91<br>89	146/86<br>90	141/89<br>90
						November - December		January - February	
32	Female<br>75<br>62<br>157<br>25.2	Uncertain (>1year)<br>Atrial fibrillation<br>Hyperuricemia	No<br>Almost every day	Irbesartan (50 mg)<br>Warfarin potassium (1 mg)<br>Bisoprolol fumarate (5 mg)<br>Lansoprazole (15 mg)<br>Cilnidipine (5 mg)<br>Topiroxostat (20 mg)<br>Ramelteon (8 mg)	None	170/110<br>66	164/107<br>68	164/107<br>68	164/105<br>66
						February - March		March - April	
33	Male<br>71<br>62<br>165<br>22.8	7 year<br>Prostate cancer (post-radiation therapy)	No<br>Almost every day	None	Squash<br>120 min<br>(4 times)	145/92<br>64	150/96<br>62	147/93<br>64	142/94<br>58
						February - March		March - April	
34	Female<br>46<br>65<br>167<br>23.3	This study (<1 month)<br>None	No<br>No	None	None	134/94<br>75	130/89<br>76	130/89<br>76	128/90<br>79
						February - March		March - April	
35	Female<br>69<br>65<br>148<br>29.7	19 years<br>Hyperlipidemia	No<br>Almost every day	None	None	149/83<br>61	148/80<br>60	149/81<br>64	149/79<br>60
						February - March		March - April	

Participant #	Sex<br>Age (years)<br>Body weight (kg)<br>Height (cm)<br>BMI	Period since diagnosis or self-recognition as hypertension<br>Declared health problems and diseases other than hypertension	Smoking<br>Alcohol (if yes, how often)	Current medication (dose per day)	Habitual exercise (times per week)	SBP/DBP (mm Hg) and HR (bpm) just before (left) and after (right) NOCR period (month of first - last bout of NOCR)		SBP/DBP (mm Hg) and HR (bpm) just before (left) and after (right) VOCR period (month of first - last bout of VOCR)	
36	Female<br>64<br>54<br>148<br>24.7	3 years<br>None	No<br>No	None	Walking 60 min (5 times)	141/87<br>52	132/82<br>51	132/82<br>51	127/81<br>51
						February - March		March - April	
37	Female<br>80<br>54<br>148<br>24.7	1 year<br>None	No<br>No	None	None	143/87<br>69	144/90<br>64	151/91<br>65	135/87<br>62
						March - April		May - June	
38	Male<br>56<br>77<br>170<br>26.6	2 years<br>None	Yes<br>No	None	None	156/96<br>67	152/93<br>69	152/93<br>69	144/87<br>66
						March - April		May - June	
39	Female<br>85<br>50.2<br>148<br>22.9	29 years<br>Atrial fibrillation<br>Diabetes mellitus	No<br>Occasionally	Candesartan cilexetil • Amlodipine (8 mg • 5mg)<br>Rivaroxaban (10 mg)<br>Rosuvastatin (5 mg)<br>Carvedilol (5 mg)<br>Metformin hydrochloride (500 mg)<br>Esomeprazole (20 mg)<br>Sennoside (60 mg)	None	136/82<br>83	139/85<br>82	137/85<br>80	128/77<br>79
						August - October		May - June	
40	Male<br>62<br>84<br>172<br>28.4	13 years<br>Diabetes mellitus	No<br>Occasionally	Valsartan • Amlodipine (80 mg • 5 mg)<br>Atorvastatin (10 mg)<br>Benzbromarone (25 mg)<br>Canagliflozin (100 mg)<br>Metformin hydrochloride (1000 mg)<br>Bisoprolol fumarate (5 mg)	None	147/80<br>73	152/80<br>75	ND	ND
						July - August		ND	

Supplementary Table 3 | Information of participants in the study of protocol 3. SBP/DBP (mm Hg) and HR (bpm) just before and after NOCR or VOCR are "value of the week"s. Five of 19 participants in protocol 3 were participants in protocol 2. The parenthesized participant numbers indicate their participant numbers in protocol 2 (see Supplementary Table 2). They participated in protocol 3 upon their eligibility check at least nine months after the last bout of VOCR in protocol 2.

Supplementary Video 1 | Rat treadmill running and PHM. Treadmill running at a velocity of 20 m/min (top), and PHM that generates mechanical accelerations with a peak magnitude of 1.0 × *g* at the head (bottom) are shown.

Supplementary Video 2 | Vertically oscillating chair riding (VOCR). This video was taken solely to present the system with consent from the person who volunteered to join the filming as an occupant of the chair. It is not a record of the human study described in this paper.

References

1. Carman, P.C. Fluid flow through granular beds. *Chemical Engineering Research and Design* **75**, S32-S48 (1997).
2. Xu, P. & Yu, B. Developing a new form of permeability and Kozeny–Carman constant for homogeneous porous media by means of fractal geometry. *Advances in Water Resources* **31**, 74-81 (2008).
3. Pedersen, J.A., Boschetti, F. & Swartz, M.A. Effects of extracellular fiber architecture on cell membrane shear stress in a 3D fibrous matrix. *J Biomech* **40**, 1484-1492 (2007).
4. Geer, C.P. & Grossman, S.A. Interstitial fluid flow along white matter tracts: a potentially important mechanism for the dissemination of primary brain tumors. *J Neurooncol* **32**, 193-201 (1997).
5. Kingsmore, K.M.*, et al.* MRI analysis to map interstitial flow in the brain tumor microenvironment. *APL Bioeng* **2**(2018).
6. Cserr, H.F. & Patlak, C.S. Secretion and bulk flow of interstitial fluid. in *Physiology and Pharmacology of the Blood-Brain Barrier* (ed. Bradbury, M.W.B.) 245-261 (Springer Berlin Heidelberg, Berlin, Heidelberg, 1992).
7. Yetkin, F.*, et al.* Cerebrospinal fluid viscosity: a novel diagnostic measure for acute meningitis. *South Med J* **103**, 892-895 (2010).
8. Bloomfield, I.G., Johnston, I.H. & Bilston, L.E. Effects of proteins, blood cells and glucose on the viscosity of cerebrospinal fluid. *Pediatr Neurosurg* **28**, 246-251 (1998).
9. Tarbell, J.M. & Shi, Z.-D. Effect of the glycocalyx layer on transmission of interstitial flow shear stress to embedded cells. *Biomechanics and modeling in mechanobiology* **12**, 111-121 (2013).

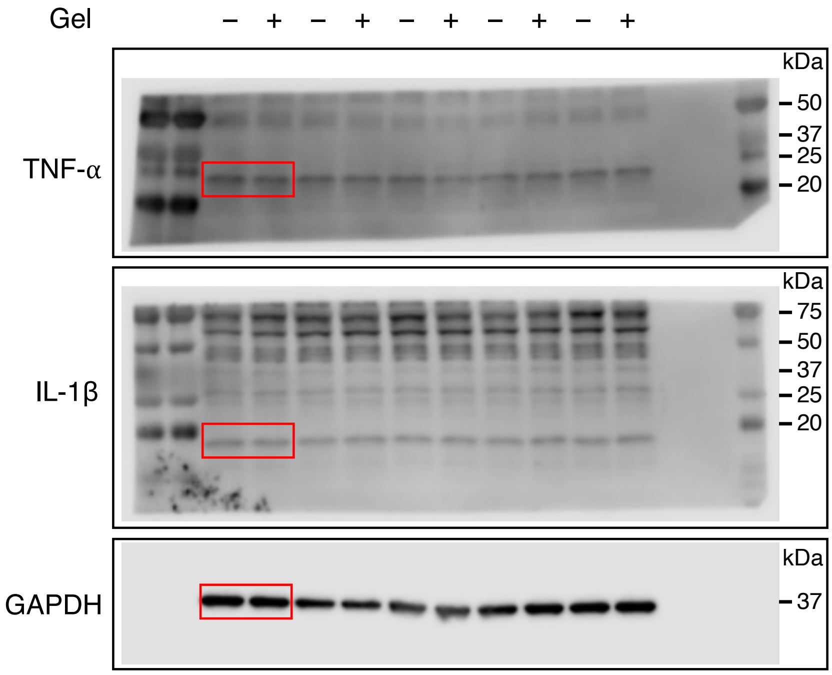

Supplementary Data 1 | Uncropped blots for Supplementary Figure 3h.

Information of Statistical Analyses

Figure	*n*	Statistical test	***P*-value**	
Figure 1b	*n* = 7 rats for each group of WKY; *n* = 8 rats for each group of SHRSP	two-way repeated measures ANOVA with Bonferroni's post hoc multiple comparisons test	SHRSP PHM (-) vs. (+) for Day15	*P* = 0.1344
			SHRSP PHM (-) vs. (+) for Day22	*P* = 0.0110
			SHRSP PHM (-) vs. (+) for Day29	*P* = 0.0463
			WKY PHM (-) vs. (+) for Day15	*P* > 0.9999
			WKY PHM (-) vs. (+) for Day22	*P* > 0.9999
			WKY PHM (-) vs. (+) for Day29	*P* > 0.9999
Figure 1c	*n* = 7 rats for each group of WKY; *n* = 8 rats for each group of SHRSP	one-way ANOVA with Tukey's post hoc multiple comparisons test	column 1 vs. 2	*P* = 0.9739
			column 3 vs. 4	*P* = 0.0046
Figure 1d	*n* = 7 rats for each group of WKY; *n* = 8 rats for each group of SHRSP	one-way ANOVA with Tukey's post hoc multiple comparisons test	column 1 vs. 2	*P* = 0.9650
			column 3 vs. 4	*P* = 0.2362
Figure 1e	*n* = 10 rats for WKY, PHM (−); *n* = 13 rats for WKY, PHM (+); *n* = 10 rats for SHRSP, PHM (−); *n* = 14 rats for SHRSP, PHM (+)	one-way ANOVA with Tukey's post hoc multiple comparisons test	column 1 vs. 2	*P* = 0.9866
			column 3 vs. 4	*P* = 0.0152
Figure 1f	*n* = 8 rats for each group of WKY; *n* = 16 rats for SHRSP, PHM (−); *n* = 13 rats for SHRSP, PHM (+)	one-way ANOVA with Tukey's post hoc multiple comparisons test	column 1 vs. 2	*P* = 0.9854
			column 3 vs. 4	*P* = 0.0085
Figure 1i	*n* = 3 rats for each group	one-way ANOVA with Tukey's post hoc multiple comparisons test	column 1 vs. 2	*P* = 0.9602
			column 1 vs. 3	*P* = 0.9215
			column 3 vs. 4	*P* = 0.9313
Figure 1j	*n* = 3 rats for each group	one-way ANOVA with Tukey's post hoc multiple comparisons test	column 1 vs. 2	*P* = 0.9455
			column 1 vs. 3	*P* = 0.0004
			column 3 vs. 4	*P* = 0.0002
Figure 2c	*n* = 5 rats for each group of WKY; *n* = 7 rats for SHRSP, PHM (−); *n* = 8 rats for	one-way ANOVA with Tukey's post hoc multiple comparisons test	column 1 vs. 2	*P* = 0.9876
			column 3 vs. 4	*P* = 0.0003

	SHRSP, PHM (+)			
Figure 2e	n = 3 rats for WKY, PHM (−); n = 5 rats for WKY, PHM (+); n = 4 rats for each group of SHRSP	one-way ANOVA with Tukey's post hoc multiple comparisons test	column 1 vs. 2	$P = 0.9953$
			column 3 vs. 4	$P = 0.0099$
Figure 3d	n = 7 rats for GFAP-AGTRAP; n = 7 rats for NSE-control; n = 6 rats for NSE-AGTRAP	two-way repeated measures ANOVA with Bonferroni's post hoc multiple comparisons test	GFAP-control vs. GFAP-AGTRAP	$P = 0.0222$
			NSE-control vs. NSE-AGTRAP	$P > 0.9999$
Figure 3e	n = 7 rats for GFAP-AGTRAP; n = 7 rats for NSE-control; n = 6 rats for NSE-AGTRAP	one-way ANOVA with Tukey's post hoc multiple comparisons test	column 1 vs. 2	$P = 0.0229$
			column 3 vs. 4	$P = 0.6864$
Figure 3f	n = 4 rats for each group	one-way ANOVA with Tukey's post hoc multiple comparisons test	column 1 vs. 2	$P = 0.0497$
			column 3 vs. 4	$P = 0.7455$
Figure 4d	n = 4 rats for each group	unpaired two-tailed Student's t-test		$P = 0.0089$
Figure 4h	n = 3 rats for each group	unpaired two-tailed Student's t-test	left chart	$P = 0.6666$
			middle chart	$P = 0.0218$
			right chart	$P = 0.0244$
Figure 5a	$n = 4$	unpaired two-tailed Student's t-test	for 0.05 Pa	$P = 0.6453$
			for 0.05 Pa	$P = 0.7517$
			for 0.3 Pa	$P = 0.0226$
			for 0.5 Pa	$P = 0.0064$
			for 0.7 Pa	$P < 0.0001$
Figure 5b	$n = 4$	unpaired two-tailed Student's t-test	for 1 mm Hg	$P = 0.5592$
			for 10 mm Hg	$P = 0.7113$
			for 20 mm Hg	$P = 0.0088$
			for 40 mm Hg	$P < 0.0001$
Figure 5d	n = 4 for 6 h FSS (−); n = 3 for the other groups	unpaired two-tailed Student's t-test	for 6 h	$P = 0.0049$
			for 24 h	P = 0. 0006
Figure 5f	n = 3 for each group	unpaired two-tailed Student's t-test	for 6 h	$P = 0.0002$
			for 24 h.	$P = 0.0104$
Figure 6b	n = 7 rats for Gel (−), PHM (+) and Gel (+),	two-way repeated measures ANOVA with Tukey's post hoc multiple comparisons test	blue vs. orange for Day 15	$P = 0.4314$
			blue vs. orange for Day 22	$P = 0.4685$

	PHM (+); *n* = 6 rats for Gel (+), PHM (−)		blue vs. orange for Day 29	*P* = 0.0389
			blue vs. black for Day 15	*P* = 0.5372
			blue vs. black for Day 22	*P* = 0.2000
			blue vs. black for Day 29	*P* = 0.0406
			black vs. orange for Day 15	*P* = 0.9911
			black vs. orange for Day 22	*P* = 0.8222
			black vs. orange for Day 29	*P* = 0.9965
Figure 6c	*n* = 7 rats for Gel (−), PHM (+) and Gel (+), PHM (+); *n* = 6 rats for Gel (+), PHM (−)	one-way ANOVA with Tukey's post hoc multiple comparisons test	column 1 vs. 2	*P* = 0.0387
			column 1 vs. 3	*P* = 0.0372
			column 2 vs. 3	*P* = 0.9959
Figure 6d	*n* = 5 rats for each group	one-way ANOVA with Tukey's post hoc multiple comparisons test	column 1 vs. 2	*P* = 0.0247
			column 1 vs. 3	*P* = 0.0307
			column 2 vs. 3	*P* = 0.9920
Figure 6f	*n* = 4 rats for each group	one-way ANOVA with Tukey's post hoc multiple comparisons test	column 1 vs. 2	*P* < 0.0001
			column 1 vs. 3	*P* < 0.0001
			column 2 vs. 3	*P* = 0.1597
Figure 6g	*n* = 4 rats for each group	one-way ANOVA with Tukey's post hoc multiple comparisons test	column 1 vs. 2	*P* = 0.6029
			column 1 vs. 3	*P* = 0.9963
			column 2 vs. 3	*P* = 0.5552
Figure 7a	*n* = 15	paired two-tailed Student's t-test	SBP	*P* = 0.0005
			DBP	*P* = 0.0011
			MAP	*P* = 0.0008
			HR	*P* = 0.7845
Figure 7b	*n* = 15 for Pre and Weeks 1 to 5; *n* = 13 for Week 6; *n* = 12 for Week 7; *n* = 11 for Weeks 8 and 9; *n* = 9 for Week 10; *n* = 7 for Week 11; *n* = 3 for Week 12	paired two-tailed Student's t-test	SBP for Pre vs. Week 1	*P* = 0.0293
			SBP for Pre vs. Week 2	*P* = 0.0028
			SBP for Pre vs. Week 3	*P* = 0.0013
			SBP for Pre vs. Week 4	*P* = 0.0035
			SBP for Pre vs. Week 5	*P* = 0.0002
			SBP for Pre vs. Week 6	*P* = 0.0078
			SBP for Pre vs. Week 7	*P* = 0.0035
			SBP for Pre vs. Week 8	*P* = 0.0075
			SBP for Pre vs. Week 9	*P* = 0.2132

			SBP for Pre vs. Week 10	$P = 0.1314$
			SBP for Pre vs. Week 11	$P = 0.0973$
			SBP for Pre vs. Week 12	$P = 0.3993$
			DBP for Pre vs. Week 1	$P = 0.3022$
			DBP for Pre vs. Week 2	$P = 0.0436$
			DBP for Pre vs. Week 3	$P = 0.0010$
			DBP for Pre vs. Week 4	$P = 0.0100$
			DBP for Pre vs. Week 5	$P = 0.0006$
			DBP for Pre vs. Week 6	$P = 0.0599$
			DBP for Pre vs. Week 7	$P = 0.0488$
			DBP for Pre vs. Week 8	$P = 0.0096$
			DBP for Pre vs. Week 9	$P = 0.9346$
			DBP for Pre vs. Week 10	$P = 0.7850$
			DBP for Pre vs. Week 11	$P = 0.0769$
			DBP for Pre vs. Week 12	$P = 0.3137$
			MAP for Pre vs. Week 1	$P = 0.1075$
			MAP for Pre vs. Week 2	$P = 0.0132$
			MAP for Pre vs. Week 3	$P = 0.0008$
			MAP for Pre vs. Week 4	$P = 0.0063$
			MAP for Pre vs. Week 5	$P = 0.0003$
			MAP for Pre vs. Week 6	$P = 0.0251$
			MAP for Pre vs. Week 7	$P = 0.0136$
			MAP for Pre vs. Week 8	$P = 0.0071$
			MAP for Pre vs. Week 9	$P = 0.6795$
			MAP for Pre vs. Week 10	$P = 0.4295$
			MAP for Pre vs. Week 11	$P = 0.0704$
			MAP for Pre vs. Week 12	$P = 0.3662$
			HR for Pre vs. Week 1	$P = 0.6287$
			HR for Pre vs. Week 2	$P = 0.7840$
			HR for Pre vs. Week 3	$P = 0.1573$
			HR for Pre vs. Week 4	$P = 0.5380$
			HR for Pre vs. Week 5	$P = 0.7331$
			HR for Pre vs. Week 6	$P = 0.6995$

			HR for Pre vs. Week 7	$P = 0.9110$
			HR for Pre vs. Week 8	$P = 0.9875$
			HR for Pre vs. Week 9	$P = 0.5866$
			HR for Pre vs. Week 10	$P = 0.9566$
			HR for Pre vs. Week 11	$P = 0.6487$
			HR for Pre vs. Week 12	$P = 0.9905$
Figure 8a	*n* = 19 for NOCR; *n* = 14 for VOCR	paired two-tailed Student's t-test	NOCR, SBP	$P = 0.9148$
			NOCR, DBP	$P = 0.6597$
			NOCR, MAP	$P = 0.7502$
			NOCR, HR	$P = 0.9002$
			VOCR, SBP	$P = 0.0001$
			VOCR, DBP	$P = 0.0051$
			VOCR, MAP	$P = 0.0006$
			VOCR, HR	$P = 0.0867$
Figure 8b	*n* = 10 for NOCR; *n* = 12 for VOCR	Wilcoxon signed-rank test	NOCR	$P = 0.492$
			VOCR	$P = 0.016$
Figure 8c	*n* = 12 for NOCR; *n* = 12 for VOCR	Wilcoxon signed-rank test	NOCR	$P = 0.969$
			VOCR	$P = 0.063$

**Figure**	***n***	**Statistical test**	***P*-value**	
Extended Data Figure 1a	*n* = 6 rats for no daily anesthesia; *n* = 8 rats for PHM (−)	two-way repeated measures ANOVA with Bonferroni's post hoc multiple comparisons test	for Day 15	$P > 0.9999$
			for Day 22	$P > 0.9999$
			for Day 29	$P > 0.9999$
Extended Data Figure 1c	*n* = 15 rats for PHM (−); *n* = 15 rats for PHM (+)	long-rank test		$P = 0.1179$
Extended Data Figure 1d	*n* = 9 rats for PHM (−); *n* = 8 rats for PHM (+)	long-rank test		$P = 0.0093$
Extended Data Figure 2b	*n* = 8 rats for PHM (−) and PHM (dorsal-ventral); *n* = 5 rats for PHM (rostral-caudal) and PHM (left-right)	two-way repeated measures ANOVA with Dunnett's post hoc multiple comparisons test	orange vs. blue for Day 15	$P = 0.0376$
			orange vs. blue for Day 22	$P = 0.0018$
			orange vs. blue for Day 29	$P = 0.0105$
			orange vs. magenta for Day 15	$P = 0.4131$
			orange vs. magenta for Day 22	$P = 0.2448$
			orange vs. magenta for Day 29	$P = 0.0148$
			orange vs. green for Day 15	$P = 0.4869$

			orange vs. green for Day 22	*P* = 0.6439
			orange vs. green for Day 29	*P* = 0.9273
Extended Data Figure 2c	*n* = 8 rats for PHM (−) and PHM (dorsal-ventral); *n* = 5 rats for PHM (rostral-caudal) and PHM (left-right)	one-way ANOVA with Tukey's post hoc multiple comparisons test	column 1 vs. 2	*P* = 0.0363
			for column 1 vs. 3	*P* = 0.0465
			for column 1 vs. 4	*P* = 0.9576
			for column 2 vs. 3	*P* = 0.9948
Extended Data Figure 3b	*n* = 8 rats for PHM (−) and PHM (2 Hz); *n* = 5 rats for PHM (0.5 Hz) and PHM (0.2 Hz)	two-way repeated measures ANOVA with Dunnett's post hoc multiple comparisons test	orange vs. blue for Day 15	*P* = 0.1871
			orange vs. blue for Day 22	*P* = 0.0519
			orange vs. blue for Day 29	*P* = 0.0282
			orange vs. magenta for Day 15	*P* = 0.7252
			orange vs. magenta for Day 22	*P* = 0.2053
			orange vs. magenta for Day 29	*P* = 0.0208
			orange vs. green for Day 15	*P* = 0.9212
			orange vs. green for Day 22	*P* = 0.9356
			orange vs. green for Day 29	*P* = 0.8950
Extended Data Figure 3c	*n* = 8 rats for PHM (−) and PHM (2 Hz); *n* = 5 rats for PHM (0.5 Hz) and PHM (0.2 Hz)	one-way ANOVA with Tukey's post hoc multiple comparisons test	for column 1 vs. 2	*P* = 0.0435
			for column 1 vs. 3	*P* = 0.0174
			for column 1 vs. 4	*P* = 0.9068
			for column 2 vs. 3	*P* = 0.8682
Extended Data Figure 3d	*n* = 8 rats for PHM (−), PHM (1 × g), and PHM (0.2 × g); *n* = 7 rats for PHM (0.5 × g)	two-way repeated measures ANOVA with Dunnett's post hoc multiple comparisons test	orange vs. blue for Day 15	*P* = 0.1812
			orange vs. blue for Day 22	*P* = 0.0504
			orange vs. blue for Day 29	*P* = 0.0274
			orange vs. magenta for Day 15	*P* = 0.4146
			orange vs. magenta for Day 22	*P* = 0.1740
			orange vs. magenta for Day 29	*P* = 0.0036
			orange vs. green for Day 15	*P* = 0.9996
			orange vs. green for Day 22	*P* = 0.9267
			orange vs. green for Day 29	*P* = 0.1618
Extended Data Figure 3e	*n* = 8 rats for PHM (−), PHM (1 × g), and PHM (0.2 × g); *n* = 7 rats for PHM (0.5 × g)	one-way ANOVA with Tukey's post hoc multiple comparisons test	column 1 vs. 2	*P* = 0.0227
			column 1 vs. 3	*P* = 0.0014
			column 1 vs. 4	*P* = 0.1664
			column 2 vs. 3	*P* = 0.6283
Extended Data Figure 4b	*n* = 3 rats for treadmill (−); *n* = 4 rats for treadmill (+)	unpaired two-tailed Student's t-test		*P* = 0.7056

Extended Data Figure 4c	*n* = 3 rats for treadmill (−); *n* = 4 rats for treadmill (+)	unpaired two-tailed Student's t-test		*P* = 0.0048
Extended Data Figure 5c	*n* = 3 rats for each group	unpaired two-tailed Student's t-test	column 1 vs. 2	*P* < 0.0001
			column 3 vs. 4	*P* < 0.0001
Extended Data Figure 5e	*n* = 3 rats for each group	unpaired two-tailed Student's t-test	column 1 vs. 2	*P* < 0.0001
			column 3 vs. 4	*P* < 0.0001
Extended Data Figure 5f	*n* = 6 rats for no AAV injection; *n* = 6 rats for GFAP-control; *n* = 7 rats for NSE-control	two-way repeated measures ANOVA with Tukey's post hoc multiple comparisons test	blue vs. orange	*P* = 0.9918
			blue vs. black	*P* = 0.6465
			orange vs. black	*P* = 0.5966
Extended Data Figure 5g	GFAP-control: *n* = 6 rats for Day 1, Day 8, and Day 15; *n* = 3 rats for Day 22 and Day 29. GFAP-AGTRAP: *n* = 7 rats for Day 1, Day 8, and Day 15; *n* = 3 rats for Day 22 and Day 29	two-way repeated measures ANOVA with Bonferroni's post hoc multiple comparisons test	Day 8	*P* = 0.5615
			Day 15	*P* = 0.0284
			Day 22	*P* > 0.9999
			Day 29	*P* > 0.9999
Extended Data Figure 8b	*n* = 6 for 6 h; *n* = 3 for 24 h/FSS (−); *n* = 6 for 24 h/FSS (+)	unpaired two-tailed Student's t-test	6 h	*P* = 0.0116
			24 h	*P* < 0.0001
Extended Data Figure 8c	*n* = 4 for each group	unpaired two-tailed Student's t-test	6 h	*P* = 0.6065
			24 h	*P* = 0.6490
Extended Data Figure 8e	*n* = 3	unpaired two-tailed Student's t-test		*P* = 0.2308
Extended Data Figure 9c	*n* = 4 rats for PHM (–); *n* = 3 rats for PHM (+)	unpaired two-tailed Student's t-test		*P* = 0.0064
Extended Data Figure 9e	*n* = 4 rats for PHM (–); *n* = 3 rats for PHM (+)	unpaired two-tailed Student's t-test		*P* = 0.0022
Extended Data Figure 10b	*n* = 8 rats for each group	two-way repeated measures ANOVA with Bonferroni's post hoc multiple comparisons test	Day 15	*P* > 0.9999
			Day 22	*P* > 0.9999
			Day 29	*P* > 0.9999
Extended Data Figure 10c	*n* = 8 rats for each group	unpaired two-tailed Student's t-test		*P* = 0.9167
Extended Data Figure 10e	*n* = 3 rats for each group	unpaired two-tailed Student's *t*-test		*P* = 0.0173
Extended Data Figure 10f	*n* = 3 rats for each group	unpaired two-tailed Student's *t*-test		*P* = 0.7812

**Figure**	***n***	**Statistical test**	***P*-value**	
Supplementary Figure 1d	*n* = 3 rats for each group	unpaired two-tailed Student's t-test		*P* = 0.8226
Supplementary Figure 1f	*n* = 3 rats for each group	unpaired two-tailed Student's t-test		*P* = 0.2342

Supplementary Figure 2b	*n* = 7 rats for treadmill (–)/Gel (–); *n* = 6 rats for treadmill (+)/Gel (–); *n* = 4 rats for each group of Gel (+)	two-way repeated measures ANOVA with Bonferroni's post hoc comparisons test	black vs. blue for Day 15	$P > 0.9999$
			black vs. blue for Day 22	$P = 0.0001$
			black vs. blue for Day 29	$P = 0.0004$
			gray vs. orange for Day 15	$P > 0.9999$
			gray vs. orange for Day 22	$P > 0.9999$
			gray vs. orange for Day 29	$P > 0.9999$
Supplementary Figure 2c	*n* = 7 rats for treadmill (–)/Gel (–); *n* = 6 rats for treadmill (+)/Gel (–); *n* = 4 rats for each group of Gel (+)	one-way ANOVA with Tukey's post hoc multiple comparisons test	column 1 vs. 2	$P = 0.0015$
			column 3 vs. 4	$P = 0.9997$
			column 1 vs. 3	$P = 0.7751$
Supplementary Figure 3b	*n* = 3 rats for each group	unpaired two-tailed Student's t-test		$P = 0.6518$
Supplementary Figure 3c	*n* = 3 rats for each group	unpaired two-tailed Student's t-test		$P = 0.6943$
Supplementary Figure 3d	*n* = 3 rats for each group	unpaired two-tailed Student's t-test		$P = 0.7938$
Supplementary Figure 3e	*n* = 3 rats for each group	unpaired two-tailed Student's *t*-test		$P = 0.8722$
Supplementary Figure 3f	*n* = 3 rats for each group	unpaired two-tailed Student's *t*-test		$P = 0.1679$
Supplementary Figure 3g	*n* = 3 rats for each group	unpaired two-tailed Student's *t*-test		$P = 0.7247$
Supplementary Figure 3h	*n* = 5 rats for each group	unpaired two-tailed Student's t-test	for TNF-α	$P = 0.2312$
			for IL-1β	$P = 0.9342$
Supplementary Figure 3i	*n* = 5 rats for each group	unpaired two-tailed Student's t-test		$P = 0.8433$
Supplementary Figure 4b	*n* = 5 rats	one-way repeated measures ANOVA with Dunnett's multiple comparisons test	SBP for beat 1	$P = 0.5829$
			SBP for beat 2	$P = 0.9994$
			SBP for beat 3	$P = 0.9627$
			SBP for beat 4	$P = 0.4169$
			SBP for beat 5	$P = 0.6948$
			SBP for beat 6	$P = 0.9999$
			SBP for beat 7	$P = 0.8152$
			SBP for beat 8	$P = 0.1381$
			SBP for beat 9	$P = 0.3191$
			SBP for beat 10	$P = 0.5286$
			MAP for beat 1	$P = 0.9906$
			MAP for beat 2	$P > 0.9999$
			MAP for beat 3	$P = 0.9997$
			MAP for beat 4	$P = 0.9997$
			MAP for beat 5	$P = 0.9349$

			MAP for beat 6	$P = 0.3562$
			MAP for beat 7	$P = 0.2239$
			MAP for beat 8	$P = 0.1599$
			MAP for beat 9	$P = 0.3001$
			MAP for beat 10	$P = 0.2596$
			DBP for beat 1	$P = 0.9810$
			DBP for beat 2	$P > 0.9999$
			DBP for beat 3	$P > 0.9999$
			DBP for beat 4	$P > 0.9999$
			DBP for beat 5	$P = 0.9996$
			DBP for beat 6	$P = 0.9997$
			DBP for beat 7	$P = 0.9421$
			DBP for beat 8	$P = 0.5327$
			DBP for beat 9	$P = 0.4949$
			DBP for beat 10	$P = 0.5215$
			HR for beat 1	$P = 0.8547$
			HR for beat 2	$P = 0.7825$
			HR for beat 3	$P =0.8267$
			HR for beat 4	$P = 0.6800$
			HR for beat 5	$P = 0.9996$
			HR for beat 6	$P = 0.9834$
			HR for beat 7	$P = 0.8658$
			HR for beat 8	$P = 0.9460$
			HR for beat 9	$P = 0.4602$
			HR for beat 10	$P =0.8992$
			ADNA for beat 1	$P = 0.6663$
			ADNA for beat 2	$P = 0.9996$
			ADNA for beat 3	$P = 0.9258$
			ADNA for beat 4	$P = 0.8960$
			ADNA for beat 5	$P = 0.9211$
			ADNA for beat 6	$P = 0.4492$
			ADNA for beat 7	$P = 0.3520$
			ADNA for beat 8	$P =0.9995$

			ADNA for beat 9	*P* =0.9979
			ADNA for beat 10	*P* = 0.5181
Supplementary Figure 5d	*n* = 5	paired two-tailed Student's t-test	SBP	*P* = 0.0018
			DBP	*P* = 0.1509
			MAP	*P* = 0.0459
			HR	*P* = 0.3900
Supplementary Figure 6b	*n* = 15	paired two-tailed Student's t-test	Aldosterone	*P* = 0.6265
			Renin activity	*P* = 0.3794
			Epinephrine	*P* = 0.5103
			Norepinephrine	*P* = 0.2653
			Dopamine	*P* > 0.9999
			CRP	*P* = 0.4412
Supplementary Figure 7b	*n* = 15 for Pre and Weeks 1 to 5; *n* = 13 for Week 6; *n* = 12 for Week 7; *n* = 11 for Weeks 8 and 9; *n* = 9 for Week 10; *n* = 7 for Week 11; *n* = 3 for Week 12	paired two-tailed Student's t-test	Male, SBP	*P* = 0.0033
			Male, DBP	*P* = 0.0094
			Male, MAP	*P* = 0.0046
			Male, HR	*P* = 0.4373
			Female, SBP	*P* = 0.0002
			Female, DBP	*P* = 0.0005
			Female, MAP	*P* = 0.0003
			Female, HR	*P* = 0.6257
