# Supplementary material for: Interstitial-fluid shear stresses induced by vertically oscillating head motion lower blood pressure in hypertensive rats and humans
Source: Nat Biomed Eng. 2023 Jul 6;7(11):1350–73. doi: 10.1038/s41551-023-01061-x (PMC10651490; doi:10.1038/s41551-023-01061-x)
Supplement: Supplementary file 2 — Reporting Summary [file 41551_2023_1061_MOESM2_ESM.pdf]

nature portfolio

Corresponding author(s): Yasuhiro Sawada

Last updated by author(s): May 24, 2023

Reporting Summary

Nature Portfolio wishes to improve the reproducibility of the work that we publish. This form provides structure for consistency and transparency in reporting. For further information on Nature Portfolio policies, see our Editorial Policies and the Editorial Policy Checklist.

Statistics

For all statistical analyses, confirm that the following items are present in the figure legend, table legend, main text, or Methods section.

n/a	Confirmed	
☐	☒	The exact sample size (*n*) for each experimental group/condition, given as a discrete number and unit of measurement
☐	☒	A statement on whether measurements were taken from distinct samples or whether the same sample was measured repeatedly
☐	☒	The statistical test(s) used AND whether they are one- or two-sided<br>*Only common tests should be described solely by name; describe more complex techniques in the Methods section.*
☐	☒	A description of all covariates tested
☐	☒	A description of any assumptions or corrections, such as tests of normality and adjustment for multiple comparisons
☐	☒	A full description of the statistical parameters including central tendency (e.g. means) or other basic estimates (e.g. regression coefficient) AND variation (e.g. standard deviation) or associated estimates of uncertainty (e.g. confidence intervals)
☐	☒	For null hypothesis testing, the test statistic (e.g. *F*, *t*, *r*) with confidence intervals, effect sizes, degrees of freedom and *P* value noted<br>*Give P values as exact values whenever suitable.*
☒	☐	For Bayesian analysis, information on the choice of priors and Markov chain Monte Carlo settings
☒	☐	For hierarchical and complex designs, identification of the appropriate level for tests and full reporting of outcomes
☒	☐	Estimates of effect sizes (e.g. Cohen's *d*, Pearson's *r*), indicating how they were calculated

Our web collection on statistics for biologists contains articles on many of the points above.

Software and code

Policy information about availability of computer code

Data collection	Microsoft Excel (Version 16.65, Microsoft, Redmond, WA), LabChart (Version 8, ADInstruments, Bella Vista, Australia), Spike2 software (Version 7.20, Cambridge Electronic Design, Cambridge, UK).
Data analysis	Prism software (Version 8, GraphPad Software, San Diego, CA), R software (Version 4.1.2, https://www.r-project.org/), MATLAB (Version 2021a, Math Works, Natic, MA). The MATLAB scripts to compute the cluster-angle and aspect-ratio distributions from multiphoton microscope images (Extended Data Fig. 6c and 7c) and to compute the lower brainstem and longest centroidal line of this part of the brain (Extended Data Fig. 6d) are available at https://doi.org/10.5281/zenodo.7936475.

For manuscripts utilizing custom algorithms or software that are central to the research but not yet described in published literature, software must be made available to editors and reviewers. We strongly encourage code deposition in a community repository (e.g. GitHub). See the Nature Portfolio guidelines for submitting code & software for further information.

Data

Policy information about availability of data

All manuscripts must include a data availability statement. This statement should provide the following information, where applicable:

- Accession codes, unique identifiers, or web links for publicly available datasets
- A description of any restrictions on data availability
- For clinical datasets or third party data, please ensure that the statement adheres to our policy

The main data supporting the findings of this study are available within the paper and its Supplementary Information. The raw data generated during the study are too large to be publicly shared, yet they are available for research purposes from the corresponding author on reasonable request. The template atlas of the Fischer 344 rat brain is available at https://doi.org/10.5281/zenodo.3900544.

Research involving human participants, their data, or biological material

Policy information about studies with human participants or human data. See also policy information about sex, gender (identity/presentation), and sexual orientation and race, ethnicity and racism.

Reporting on sex and gender	We used only male rats for our animal studies (see below). However, because we did not aim to investigate only male-specific matters, we included human participants of both sexes in our clinical study. Vertically oscillating chair riding had antihypertensive effects in both male and female humans (Supplementary Fig. 7b). We list the participants' sex in Supplementary Tables 2 and 3.
Reporting on race, ethnicity, or other socially relevant groupings	The eligibility to participate in the clinical study did not involve any particular race, ethnicity, or other socially relevant groupings. However, all the applicants for this study were Japanese, and hence we did not control for race and ethnicity as possible confounding variables in our analyses.
Population characteristics	Both females and males were considered eligible if they were 20 years old or older and confirmed to have 130–160 mm Hg of systolic blood pressure at the time of interview for informed consent and eligibility check. Subjects with mental or psychological illnesses, history or presence of cardiovascular events, history or presence of severe dysfunction/disorder of liver, kidney, lung, gastrointestinal tract, and spine, or presence of acute injuries/diseases (such as recent traumas and infectious diseases) were excluded, with the exception of those who were given permission for participating in this study from their primary care physicians. Two male and three female subjects aged 37–60 years participated in the study of protocol 1. Sixteen males and 14 females aged 23–85 years participated in the studies of protocols 2 or 3. Three males and two females participated in the studies of both protocols 2 and 3.
Recruitment	For the study of protocol 1, participants were recruited through printed advertisements placed in Iwai Orthopaedic Medical Hospital and the affiliated health services facility of Iwai Medical Foundation (Iwai Keiaien). For the studies of protocols 2 and 3, participants were recruited through printed advertisements placed in the National Rehabilitation Center for Persons with Disability Hospital and the Tokorozawa Heart Center. There may be potentially self-selection bias related to the recruitment of the participants in our human studies. For example, the participants may have had more interest in their health condition as compared to ordinary people. Although this may have influenced the results of our human study, antihypertensive or sympathoinhibitory effects were not observed in non-oscillating chair riding. This supports the notion that vertically oscillating chair riding is specifically effective.
Ethics oversight	The study of protocol 1 was approved by the Ethics Committee of the Iwai Medical Foundation, and the studies of protocols 2 and 3 were approved by the Ethics Committee of the National Rehabilitation Center for Persons with Disabilities. All participants in our human studies provided written informed consent.

Note that full information on the approval of the study protocol must also be provided in the manuscript.

Field-specific reporting

Please select the one below that is the best fit for your research. If you are not sure, read the appropriate sections before making your selection.

☒ Life sciences ☐ Behavioural & social sciences ☐ Ecological, evolutionary & environmental sciences

For a reference copy of the document with all sections, see nature.com/documents/nr-reporting-summary-flat.pdf

Life sciences study design

All studies must disclose on these points even when the disclosure is negative.

Sample size	No statistical methods were used to predetermine sample size. At least three biologically independent experimental replicates were performed for statistical analyses, according to standard scientific conventions. In the case of experiments using hypertensive rats, we in principle used similar sizes to those in published experiments to analyse the effects of exercise on hypertensive rats (Kishi et al. Clin Exp Hypertens 2012; Bertagnolli et al. Am J Hypertens 2008; Agarwal et al. Hypertension 2009). We tried to reach a conclusion for each individual experiment, using the smallest sample size possible.

Data exclusions	No data were excluded.
Replication	Information on replication, or on the independent performance of the experiments and measurements, is provided in the figure legends and Methods. Unless stated otherwise, the experiments or measurements were replicated or performed at least three times independently.
Randomization	All the animals and cells were randomly assigned to experimental groups.
Blinding	Blinding was irrelevant to the study. All animal and cell experiments were carried out by researchers who prepared and analysed the samples.

Reporting for specific materials, systems and methods

We require information from authors about some types of materials, experimental systems and methods used in many studies. Here, indicate whether each material, system or method listed is relevant to your study. If you are not sure if a list item applies to your research, read the appropriate section before selecting a response.

Materials & experimental systems

n/a	Involved in the study	
☐	☒	Antibodies
☐	☒	Eukaryotic cell lines
☒	☐	Palaeontology and archaeology
☐	☒	Animals and other organisms
☒	☐	Clinical data
☒	☐	Dual use research of concern
☒	☐	Plants

Methods

n/a	Involved in the study	
☒	☐	ChIP-seq
☒	☐	Flow cytometry
☐	☒	MRI-based neuroimaging

Antibodies

Antibodies used	Mouse monoclonal anti-GFAP cloneGA5 (cloneGA5, MAB360; Millipore) ; rabbit polyclonal anti-GFAP (Z0334; Dako) ; chicken polyclonal anti-GFAP (ab4674; Abcam) ; rabbit polyclonal anti-cleaved caspase-3 (9661; Cell Signaling Technology) ; mouse monoclonal anti-NeuN (clone A69, MAB377; Millipore) ; rabbit polyclonal anti-NeuN (ABN78; Millipore) , rabbit polyclonal anti-AT1R (HPA003596; Sigma-Aldrich) ; rabbit polyclonal anti-AGTRAP (HPA044120; Sigma-Aldrich) ; rabbit polyclonal anti-GFP (598; MBL) ; chicken polyclonal anti-GFP (ab13970; Abcam) ; mouse monoclonal anti-TUJ-1 (clone 2G10, ab78078; Abcam); rabbit polyclonal anti-TNF-alpha (ab66579; Abcam) ; rabbit polyclonal anti-IL-1beta (ab9722; Abcam); rabbit polyclonal anti-GAPDH (5174; Cell Signaling Technology). Secondary antibodies conjugated with Alexa Fluor 350, 488, 568, 633, and 647 (Thermo Fisher Scientific). Horseradish peroxidase-conjugated anti-rabbit IgG (H + L) secondary antibody (W401B; Promega).
Validation	All antibodies were obtained from commercial manufacturers. Anti-AT1R antibody was validated by verifying the consistency with anti-hemagglutinin (HA) (mouse monoclonal, clone HA.C5, ab18181; Abcam) immunostaining of HA-tagged AT1R exogenously expressed in HEK293 cells, which do not express AT1R endogenously. All the other antibodies were employed based on the validation statements provided on the manufacturers' websites.

Eukaryotic cell lines

Policy information about cell lines and Sex and Gender in Research

Cell line source(s)	Neuro2A cell line (mouse neuroblastoma), which was provided by Dr. T. Yokota (Tokyo Medical and Dental University).
Authentication	Although the expression of neuronal proteins was confirmed, the cell line used was not authenticated.
Mycoplasma contamination	The cell line was not tested for mycoplasma contamination. However, when we conducted DAPI staining, we did not see a dotted stain around the nuclei, which is characteristic of mycoplasma contamination.
Commonly misidentified lines (See ICLAC register)	No commonly misidentified cell lines were used.

Animals and other research organisms

Policy information about studies involving animals; ARRIVE guidelines recommended for reporting animal research, and Sex and Gender in Research

Laboratory animals	Male SHRSP/Izm and WKY/Izm rats were provided by the Disease Model Cooperative Association (Kyoto, Japan) and used for experiments at the age of 9 to 21 weeks after acclimation for at least 1 week. The astrocyte-GFP mice (Aldh1L1-GFP mice) obtained from GENSAT and bred in-house were housed with free access to water and standard rodent chow under a 12/12 h light–dark cycle with controlled temperature (22–24°C) and humidity (50–60%). One-day-old or two-day-old mice of both genders were used for astrocyte preparation.

Wild animals	The study did not involve wild animals.
Reporting on sex	We used only male rats for our animal studies, although sex is an important variable for nearly all diseases, including hypertension. This was because we intended to preclude or minimize the potential influence of oestrogen and progesterone, both of which basically act protectively on cardiovascular systems, including the heart and endothelium. Perhaps for the same reason, in many or even most of the animal experiments in previous studies investigating the pathogenesis of cardiovascular diseases, male animals have been used unless there is particular reason to analyse female animals. In particular, we intended to be consistent with previous studies that investigated antihypertensive effects of treadmill running in male SHRs or SHRSPs.
Field-collected samples	The study did not involve samples collected from the field.
Ethics oversight	The animal study was approved by the Animal Care and Use Committee of the National Rehabilitation Center for Persons with Disabilities.

Note that full information on the approval of the study protocol must also be provided in the manuscript.

Magnetic resonance imaging

Experimental design

Design type	—
Design specifications	—
Behavioral performance measures	—

Acquisition

Imaging type(s)	Structural
Field strength	7 Tesla
Sequence & imaging parameters	2D rapid acquisition with relaxation enhancement (RARE). Parameters for the sagittal T2-RARE sequence were as follows; echo time (TE): 33 ms, repetition time (TR): 3600 ms, slice thickness (SL): 0.75 mm, field of view (FOV): 38.4 × 38.4 mm, voxel: 0.15 × 0.15 × 0.75 mm3. RARE factor:8, number of Averages: 4.
Area of acquisition	Whole brain

Diffusion MRI ☐ Used ☒ Not used

Preprocessing

Preprocessing software	SPM12 on MATLAB2021a
Normalization	We mainly used inverse normalization. First, we used the 'Old normalize' tool of SPM12 to compute individual data normalization. Then the brainstem template was inverse-normalized into each individual volume.
Normalization template	An MRI-Derived Neuroanatomical Atlas of the Fischer 344 Rat Brain (Version v4) [Data set]. Zenodo. https://doi.org/10.5281/zenodo.3900544.
Noise and artifact removal	—
Volume censoring	—

Statistical modeling & inference

Model type and settings	—
Effect(s) tested	—

Specify type of analysis: ☐ Whole brain ☒ ROI-based ☐ Both

Anatomical location(s): Brainstem

Statistical type for inference (See Eklund et al. 2016)	—
Correction	—

Models & analysis

n/a	Involved in the study
☒	☐ Functional and/or effective connectivity
☒	☐ Graph analysis
☒	☐ Multivariate modeling or predictive analysis
